# Self-Learning Event Mistiming Detector Based on Central Pattern Generator

**DOI:** 10.3389/fnbot.2021.629652

**Published:** 2021-02-04

**Authors:** Rudolf Szadkowski, Miloš Prágr, Jan Faigl

**Affiliations:** Computational Robotics Laboratory, Faculty of Electrical Engineering, Czech Technical University in Prague, Prague, Czechia

**Keywords:** locomotion, central pattern generator, Hebbian learning, phase estimation, radial basis function neuron, reflexes, hexapod walking robot, bio-inspired robotics

## Abstract

A repetitive movement pattern of many animals, a gait, is controlled by the Central Pattern Generator (CPG), providing rhythmic control synchronous to the sensed environment. As a rhythmic signal generator, the CPG can control the motion phase of biomimetic legged robots without feedback. The CPG can also act in sensory synchronization, where it can be utilized as a sensory phase estimator. Direct use of the CPG as the estimator is not common, and there is little research done on its utilization in the phase estimation. Generally, the sensory estimation augments the sensory feedback information, and motion irregularities can reveal from comparing measurements with the estimation. In this work, we study the CPG in the context of phase irregularity detection, where the timing of sensory events is disturbed. We propose a novel self-supervised method for learning mistiming detection, where the neural detector is trained by dynamic Hebbian-like rules during the robot walking. The proposed detector is composed of three neural components: (i) the CPG providing phase estimation, (ii) Radial Basis Function neuron anticipating the sensory event, and (iii) Leaky Integrate-and-Fire neuron detecting the sensory mistiming. The detector is integrated with the CPG-based gait controller. The mistiming detection triggers two reflexes: the elevator reflex, which avoids an obstacle, and the search reflex, which grasps a missing foothold. The proposed controller is deployed and trained on a hexapod walking robot to demonstrate the mistiming detection in real locomotion. The trained system has been examined in the controlled laboratory experiment and real field deployment in the Bull Rock cave system, where the robot utilized mistiming detection to negotiate the unstructured and slippery subterranean environment.

## 1. Introduction

Maintaining fluent gait motion in a body with a high degree of freedom while continually reacting to terrain irregularities is a challenging problem that, however, can be observed in nature (Bekey, 1996). During the gait, the legged locomotion control sustains the regular repetitive motion using reflexive reactions triggered by detected motion irregularities. In nature, animals demonstrate stunning adaptability to motion disruptions through reflexes (Pearson and Franklin, 1984; Duysens et al., 2000). Many of such reflexes are wired in neural circuits located close to the legs inside the vertebrates' spine or thoracic ganglia of many invertebrates. The spinal neural circuits must recognize an irregularity in the locomotion through proprioception to trigger a reflex (Bekey and Tomovic, 1986). Hence, the irregularity recognition needs a model of regularity to which a measured state is compared. In this work, we focus on phase irregularities, where the timing of the measured event is compared to its estimate. The tool for phase modeling is a neural structure that centrally generates rhythms, the Central Pattern Generator (CPG).

CPGs play an essential role in gait locomotion control. The CPG's rhythmic patterns are combined with the sensory-motor neural circuits and stabilize the gait periodicity. The CPG activity and spinal neural control can generally be controlled by descending (e.g., from the brain) signals. Interestingly, the locomotion can be sustained without the brain's participation and sensory input in virtual locomotion (Brown, 1912), since the CPG sustains its rhythmic signals even if it is disconnected from its sensors and effectors. This suggests the CPG can work in an open-loop mode, and thus the CPG provides the motor control even without input excitations. On the other hand, if the CPG is synchronized to the sensory signals, the CPG acts as an estimator of the sensory phase (Kuo, 2002).

We can identify that some signals are tightly coupled to the gait motion and thus inherit the gait period, such as swing stop or ground contact. The CPG that synchronizes to such a periodic signal continually estimates the signal phase. The estimated and measured sensory phase should be the same during a regular motion. However, a regular motion disturbed by unexpected dynamics, elevations, and depressions can induce disturbances in the sensory signal. Hence the motion irregularities can be detected by comparing the measured sensory phase with its estimation (Miall and Wolpert, 1996). Any difference between the timing of the measured and estimated sensory events can be utilized for mistiming detection (Goldschmidt et al., 2014), which is insufficiently researched within the context of plastic CPG-based neural networks.

**Figure 1:**
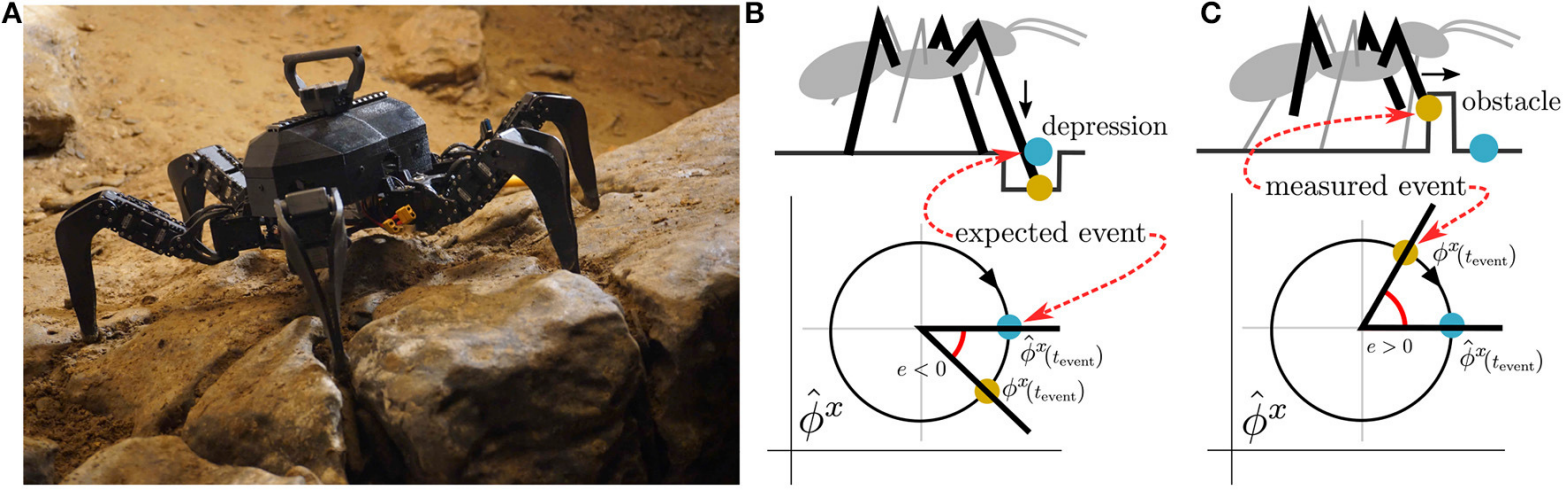
**(A)** The utilized hexapod walking robot in Bull Rock cave. The unstructured environment causes motion disturbances, which result in sensory event mistiming. The sensory phase ϕ^*x*^ measurement is compared to its estimation ${\hat{\phi }}^{x}$, where their difference is the phase error *e*. We distinguish two types of phase error: event absence, $\phi^{x}>{\hat{\phi }}^{x}$, and disruption, $\phi^{x}<{\hat{\phi }}^{x}$. An example of event absence is illustrated in **(B)**, where at the beginning of the stance phase, the front leg finds itself in a depression (orange dot) and thus detects the ground contact later than expected (blue dot). In the disruption example **(C)**, at the end of the swing phase, the front leg hits elevated terrain (orange dot) and thus detects the ground contact sooner than expected (blue dot).

In this paper, we propose a trainable CPG-based event mistiming detector integrated into gait controller architecture introduced in Szadkowski and Faigl (2020). Unlike common architectures that model the phase of sensed (input) signal and motor (output) signal with one CPG, the employed architecture models each signal with either the motor CPG, generating the motor signal phase, or sensory CPG, estimating the phase of the sensory signal. We propose to utilize the sensory CPG for the detection of irregularities in the sensory phase. We couple a plastic Radial Basis Function (RBF) neuron to each sensory CPG, which learns to anticipate sensory events. The difference in timing of anticipated and measured events is the phase error. The error is integrated by Leaky-Integrate-and-Fire (LIF) neuron, which learns to distinguish the regular phase error induced by regular measurement imperfections, and fires on irregular phase error detecting the event mistiming. Two types of event mistiming are distinguished: event absence, which occurs when the sensory event is delayed, and event disruption occurs when the sensory event is too early; see Figure 1. Both types of event mistiming are detected by the proposed CPG-based mistiming detector that augments the sensory feedback information.

We demonstrate the benefits of the proposed mistiming detector using the detection as a trigger of two reflexes: the elevator and search reflexes. The elevator reflex elevates the leg to avoid an obstacle detected during the leg swing phase. The search reflex is a behavior where the leg searches for supporting ground after not detecting the expected support at the end of the swing phase. Hence, the elevator reflex is triggered by the early stop of the swinging leg, and the escape reflex is triggered by ground contact absence. Finally, even though the focus of this work is plastic mistiming detection, we also extend the motor control of our previous work to control multiple motion phases with position and maximum torque commands.

The proposed CPG-based controller is deployed on a real hexapod walking robot. The robot is trained to walk tripod gait on flat terrain. First, the robot self-learns to estimate the sensory phase needed for mistiming detection in a regular environment. Then, we demonstrate the mistiming detector by guiding the robot over elevations and depressions in two scenarios. In the first scenario, the robot walks in a controlled environment, where the detections are isolated and thus easily observable. The second scenario tests the proposed controller's limits in the Bull Rock cave system, which provides highly unstructured terrain depicted in Figure 1A.

The rest of the paper is organized as follows. The following section is dedicated to related work. In section 3, the phase estimation problem is described within the context of gait control and the theoretical foundations for the event mistiming detection. The CPG-based controller is presented in section 4, where the sensory prediction and mistiming detection system is described, followed by the description of the motor control and reflex system. The experimental deployment is described in section 5 and further discussed in section 6. Finally, the paper is concluded in section 7.

## 2. Related Work

CPG-based gait controllers were proposed for many robots and body models, where the controller implementations vary in architecture. In this section, we provide a brief overview of existing related CPG-based controller architectures. In particular, we focus on whether the CPG represents the phase of a sensory signal (input), motor/control signal (output), or both. Existing CPG-based controllers primarily use the CPG as a generator of the motor phase. For example, the CPG in the controller presented in Maufroy et al. (2008) determines whether the leg is in the extension or flexion phase to select a subnetwork that controls the respective actuator. Similarly in limbless locomotion, a chain of coupled CPGs controls the flexion rhythm of each servomotor in a modular lamprey-like robot (Li et al., 2014). Locomotion patterns can be changed by altering the parameters of the CPG. In Yu et al. (2020), the frequency of the CPG oscillation is temporarily increased as a part of reflexive behavior, where the leg performs fast spiral motions. Switching the topology of coupling between CPGs changes the gait pattern, which is used in Wang et al. (2014) where CPG network generates multiple gaits for a fish-like robot, such as forward and backward swimming and turning. Besides the motor signal generation, a CPG can also be used as a sensory phase estimator. A CPG that is entrained by a periodic sensory signal can become synchronized with the signal where the phases of the CPG and its entraining signal evolve at the same rate (except for a short transient behavior) (Pikovsky et al., 2001). In Kuo (2002), Kuo proposes the CPG synchronization to model the sensory signal phase continuously. He showed that the actuator controller that uses the CPG's sensory estimate, is more stable than a controller using a raw sensory signal.

The difference between a motor CPG and a sensory CPG is that the former represents an actuator phase, while the latter represents a phase of the entraining sensory signal. Assuming the sensor and motor phases are the same, a single CPG can represent both phases. In Yan et al. (2017), it is assumed that the gait phase is a function of the sensory phase, e.g., a function of the hip joint angle. Thus the gait phase is estimated by the CPG synchronized to sensory events, such as maximum hip flexion. The functional dependence between the sensory and motor variables is implicitly assumed by synchronizing the CPG to the sensory input and using the same CPG as the motor phase generator (Fukuoka et al., 2003; Endo et al., 2004; Righetti and Ijspeert, 2006). However, such an architecture needs some prior knowledge about the robot morphology, where it must be determined which motors and sensors are functionally dependent. On the other hand, the morphology agnostic approach is not to assume any functional dependence and model each phase, be it sensory or motor, with its respective CPG. The controller presented in Héliot and Espiau (2008) is composed of a layer of the sensory CPGs estimating the phase that is fused and fed into the central motor CPG, which controls the gait phase. A more general approach is presented in our previous work (Szadkowski and Faigl, 2020), where both the sensory and the motor variables have their own CPGs forming a layer of sensory CPGs, which is connected to a layer of the motor CPGs. Hence, the CPGs in biomimetic controllers have two basic roles: motor phase generator and the sensory phase estimator. In the rest of this section, we focus on the sensory CPGs only, as the proposed approach enriches their utilization.

A sensory model that estimates the sensory state can help in the detection of motion disturbances. In the context of animal locomotion, such disturbances can be small obstacles, depressions, slippage, and others, to which the animal reacts with reflexes documented in Pearson and Franklin (1984) and Duysens et al. (2000). The reflexes are triggered by proprioceptive events such as increased load on a muscle or tensile sensing (Bekey and Tomovic, 1986; Duysens et al., 2000), which indicates a motion disturbance. Motion disturbance detection is implemented in a number of biomimetic reflex controllers, where each reflex has to be triggered by such a disturbance. The disturbance detection can be realized by comparing the estimated values with the measured ones; if the difference is too high, a disturbance is detected. In the context of periodic sensory signals, two differences can be measured: difference in amplitude and difference in phase. The amplitude trigger is simple; the detector directly measures a value above (or below) a certain threshold, which triggers the reflex reaction. For example, the reflexive slip responses can be triggered by detecting leg movement while the leg is on the ground (Boone and Hodgins, 1995). The elevator reflex, where the leg avoids an obstacle blocking its protraction during a swing motion, can be triggered by a significant angle error in the protractor motor, as shown in Klaassen et al. (2002). The author of Bläsing (2006) shows that the search reflex, where the leg tries to find support during the stance, can be triggered by lowering the leg under the threshold, which indicates a gap. Besides, the search and elevator reflexes are implemented in multiple other controllers (Espenschied et al., 1996; Li et al., 2018; Yu et al., 2020). However, the above-mentioned reflex triggers are hand-tuned and thus dependent on the robot body morphology. Generally, the robot morphology can change in time or is not entirely known, and thus the disturbance detection algorithm must adapt. A simple, adaptive mechanism is used in Lewinger and Quinn (2010), where the system remembers the depressor motor position during the last stance. Another learning algorithm is presented in Kirkwood et al. (1989), where the controller is trained to fuse multiple sensor inputs into a given reflex trigger.

The presented amplitude-based detectors are dependent on measuring unusual sensory values directly, where the value crosses a threshold. However, some disturbances do not change the sensory signal's amplitude but a phase, causing a sensory mistiming, such as the absence of anticipated foot contact or protraction stopping too early. The event mistiming can be detected from the difference between the phase measurement and phase estimation provided by the internal model. Generally, the internal model estimates the sensory feedback either by directly processing the current sensory measurement or processing the copy of motor command (so-called efference copy) (Miall and Wolpert, 1996). In Goldschmidt et al. (2014) the efference copy from a motor CPG is processed into a ground contact phase estimation, where the absence of ground contact triggers the search reflex. Maffei et al. pointed out that the sensory model that maps the efference copy onto sensory estimation is sensitive to the specific controller configuration. The authors propose to adapt the sensory model directly to the sensory feedback (Maffei et al., 2017). In the context of phase estimation, the CPG entrained to the sensory feedback estimates the sensory phase. The idea of phase estimating CPGs introduced in Kuo (2002) is expanded in Dzeladini et al. (2014), where the difference between the measured and estimated sensory phase is used as a corrective term that participates in motor activity regulation. However, the authors use one CPG per actuator and select the entraining sensory feedback using prior knowledge.

In the proposed approach, we leverage the sensory/motor CPG distinction presented in Szadkowski and Faigl (2020) and design a self-learning mistiming detector on the sensory CPG layer. Hence, the main expected advantage of the proposed motion irregularity detection is that no prior knowledge about sensory-motor relation is needed.

## 3. Problem Statement

**Figure 2:**
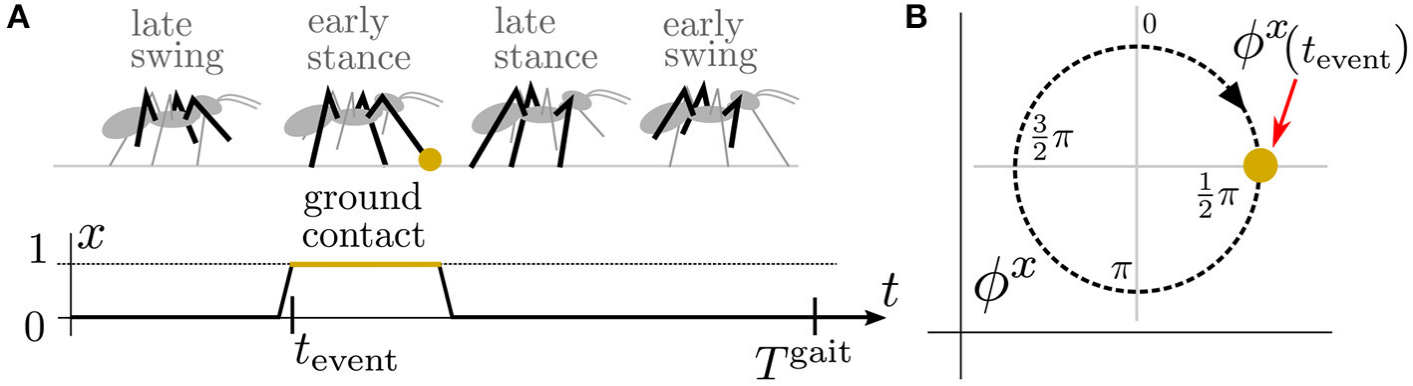
**(A)** An illustration of an ant during the tripod gait, a motion pattern where three legs propel the body while the other three legs swing forward. During the tripod gait, the ant puts a front leg on the ground and senses the ground contact with *x*(*t*_event_) = 1 at the fourth of the gait period $t_{\text{event}}=\frac{1}{4}T^{\text{gait}}$. During the regular motion, such an event occurs periodically with $x\left(t_{\text{event}}+nT^{\text{gait}}\right)=1$ for any n∈N. Therefore, **(B)** for the sensory signal *x*, we define the sensory phase ϕ^*x*^ on which we can map the event occurrence at $\phi^{x}\left(t_{\text{event}}+nT^{\text{gait}}\right)=\frac{1}{2}\pi$ for any n∈N. Notice that the sensory phase is directly measured only at *t*_event_, and there is no sensory phase measurement for the rest of the gait cycle.

The sensory mistiming detection is based on the periodicity of the sensory signal, which is entrained by the repetitive gait motion. The repetitive motion pattern arises from the rhythmical motor actuation. The motor actuation is controlled by the *control signal* **u**(*t*) which has period *T*^g*ait*^ during the regular motion. The periodically actuated body interacts with the environment, and the effects of the interactions are measured by sensors. We focus on such a *sensory signal* **x**(*t*) that inherits the actuation periodicity *T*^g*ait*^. The motor ϕ^**u**^ and sensory ϕ^**x**^ phases are defined as variables that grow linearly with time at the rate ω^g*ait*^ = 2π(*T*^g*ait*^)^−1^ during the regular motion, formally $\dot{\phi^{\text{x}}}=\dot{\phi^{\text{u}}}=\omega^{\text{g}ait}$; see Figure 2. Likewise, we define the sensory amplitude *A*^**x**^ as a variable that does not change, i.e., Ȧ^**x**^ = 0 and similarly for the motor amplitude *A*^**u**^; however, this work is focused on the phase variables.

The *phase difference* between sensory and motor phases Δϕ^**ux**^ = ϕ^**u**^(*t*) − ϕ^**x**^(*t*) is not changing in regular environments with $\Delta {\dot{\phi }}^{\text{ux}}={\dot{\phi }}^{\text{u}}-{\dot{\phi }}^{\text{x}}=0$, but it is dynamic in irregular environments, which cause disturbance of the motion. The motion disturbances propagate into the controller through the sensory signal, and the controller needs to react to sustain the regular gait.

The disturbance in a sensory signal can be assessed by comparing the sensory signal with the *sensory estimation* $\hat{\text{x}}\left(t\right)$. Focusing on the phase, the *sensory phase estimation* ${\hat{\phi }}^{\text{x}}\left(t\right)$ yields the phase of a sensory signal during regular motion: ${\hat{\phi }}^{\text{x}}\left(t\right)=\omega^{\text{g}ait}t+\Phi$, where Φ is the sensory phase at *t* = 0. During the regular motion, the phase difference between estimated and measured phase, refered to as *phase error*, is $e\left(t\right)=\phi^{\text{x}}\left(t\right)-{\hat{\phi }}^{\text{x}}\left(t\right)=0$. However, the phase error can be non-zero due to sensory signal disturbances caused by irregular motion. The authors of Pikovsky et al. (2001) describe the disturbance in dynamic systems with stable periodicity as perturbations in the phase and amplitude of the system. The perturbations can be approximately formalized as $\dot{A^{\text{x}}}\left(t\right)=p^{A}\left(t\right)$ and ${\dot{\phi }}^{\text{x}}\left(t\right)=\omega^{\text{g}ait}+p^{\phi }\left(t\right)$, where *p*^*A*^(*t*) and *p*^ϕ^(*t*) are amplitude and phase perturbations, respectively. The phase error then gains dynamics driven by the phase perturbation ė(*t*) = ω^g*ait*^ + *p*^ϕ^(*t*) − ω^g*ait*^ = *p*^ϕ^(*t*). Hence, the positive error *e*(*t*) > 0 represents sensory signal being ahead of time while negative *e*(*t*) < 0 is being delayed, which is illustrated in Figures 1B,C. If the phase error accumulated over one gait cycle exceeds a given threshold, $\int_{\tau -T^{\text{gait}}}^{\tau }|e\left(t\right)|dt>\theta$, then *the sensory mistiming* is detected at the time τ.

There are two necessary tools for detecting the sensory mistiming: the sensory phase estimator ${\hat{\phi }}^{\text{x}}\left(t\right)$ and the phase error threshold θ. Moreover, the sensory phase is rarely measured continually, as pointed out in Héliot and Espiau (2008). Instead, it is measured as a short periodic event, and only during this sensory event, the phase measurement can be compared to the phase estimation. In this work, the *i*-th sensory input *x*_*i*_(*t*) ∈ [0, 1] is a binary signal, where its high level *x*_*i*_(*t*) ≈ 1 indicates the *event*. However, since each sensor has a different sensitivity and the sensory events have different duration, the estimator and the error threshold must be self-learned for each sensor input. The proposed neurodynamic approach for self-learnable mistiming detection and its utilization in gait locomotion is presented in the next section.

## 4. The Gait Locomotion Controller

**Figure 3:**
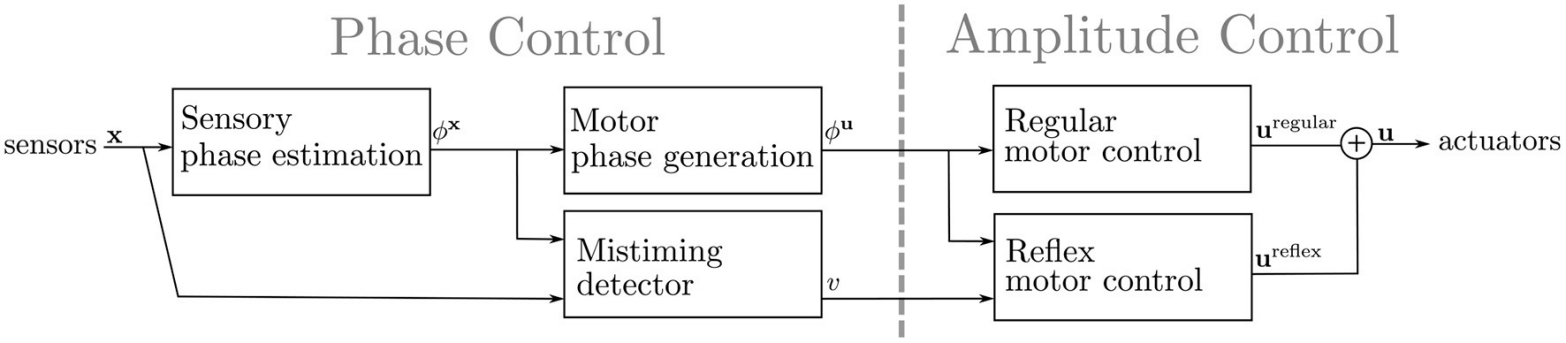
The proposed gait controller architecture takes the sensory signal *x* as the input and outputs the control signal *u*. The gait controller is composed of two sub-controllers: (i) Phase Control, which detects the mistiming and regulates the phase of the gait, and (ii) Amplitude Control, which maps the motor phase ϕ^*u*^ and mistiming detections *v* into actuator commands *u*. The phase control is CPG-based, where a coupled ensemble of CPGs estimates the sensory phase ϕ^*x*^ and generates the motor phase ϕ^*u*^. The mistiming detector compares the sensory phase estimation ϕ^*x*^ to sensory input *x*, and self-learns to detect sensory phase errors *v*. The mistiming detection *v* and generated motor phase ϕ^*u*^ flow into the amplitude control, which transforms the inputs into the control signal *u*. There are two modules of the amplitude control: the regular control and the reflex control that modifies the regular control if triggered by mistiming detection.

This section presents the proposed sensory event mistiming detector that is integrated within the CPG-based gait controller. The overall architecture of the gait controller, depicted in Figure 3, can be described as two coupled sub-controllers: the *phase control*, which estimates the phase of sensory input and generates the motor phase, and the *amplitude control*, which generates the command values for the actuators. The phase controller is composed of two CPG layers: the *sensory CPG*s that estimate the phase for each *i*-th sensory input $\phi_{i}^{\text{x}}$, and the *motor CPG*s that generate the motor phase of each *j*-th actuator $\phi_{j}^{\text{u}}$. The sensory CPGs provide a continuous estimation of the sensory input phases utilized by the motor CPG. The motor CPGs generate the phase of the motion for each actuator. Based on the motor phase, the amplitude control generates the control signal **u**_*j*_ for each *j*-th actuator, which performs the regular motion. In this work, the amplitude control is extended with reflex reactions to motion disturbances triggered by mistiming detection. The mistiming detector is an extension of the sensory CPG layer utilizing the provided sensory phase estimation.

### 4.1. Central Pattern Generator as Phase Estimator

The CPG provides a stable periodic rhythm that can be synchronized with an input signal. In the gait motion context, the periodic stability sustains the motion periodicity while the synchronization is utilized for the sensory phase estimation. The synchronization is a property of CPGs modeled as a dynamic system with a limit-cycle attractor (Pikovsky et al., 2001). The employed CPG can be formalized as follows.

Let ẏ = *f*(**y**, *c*(*t*)) ∈ ℝ^*D*^ be the CPG dynamics in the *D*-dimensional space with the *input signal* *c*(*t*). The *limit-cycle* **Y** ⊂ ℝ^*D*^ is a closed trajectory in the phase space to which the unperturbed dynamic system **y**(*t*) converges. After the convergence, the unperturbed CPG produces a stable periodic signal with the *natural frequency* ω^cpg^. If the CPG is entrained by the periodic signal *c*(*t*) with a frequency close to the natural frequency ω ≈ ω^cpg^, the CPG synchronizes the input signal. The synchronization is a phase relation, where the phase difference between the CPG output and the entraining signal Δϕ^**y***c*^ = ϕ^**y**^(*t*) − ϕ^*c*^(*t*) becomes stable. Note that the stable phase difference implies that the entrained CPG frequency becomes the same as the entraining signal frequency ω^cpg^ = ω, and if the phase of the input signal shifts, the phase of the CPG shifts as well. Hence, the phase of the synchronized CPG continuously estimates the phase of the entraining signal: ${\hat{\phi }}^{c}\left(t\right)=\phi^{\text{y}}\left(t\right)-\Delta \phi^{\text{y}c}$. However, since neither the phase difference Δϕ^**y***c*^, nor the function that maps the CPG state **y** ∈ **Y** onto the CPG phase ϕ^**y**^(*t*) are known in general, the explicit value of the CPG phase ϕ^**y**^(*t*) cannot be directly used in practice. Instead, we exploit the fact that there exists one-to-one mapping between the CPG phase ϕ^**y**^(*t*) ∈ [0, 2π) and the limit-cycle points **Y**(ϕ^**y**^) = **y**. Thus, since $\text{Y}\left(\phi^{\text{y}}-\Delta \phi^{\text{y}c}\right)=\text{Y}\left({\hat{\phi }}^{c}\right)$ is one-to-one mapping, each point on the limit-cycle **y** ∈ **Y** represents the phase of the entraining signal ${\hat{\phi }}^{c}$. This limit-cycle representation of the input signal phase is the essential CPG property in the proposed approach.

We employ Matsuoka's neural oscillator (Matsuoka, 1987) as the CPG

(1) $$\begin{array}{l} ẏ=f\left(\text{y},c\left(t\right)\right)=\left[\begin{array}{c} \tau {ẏ}_{1}\\ \tau {ẏ}_{2}\\ \gamma {ẏ}_{3}\\ \gamma {ẏ}_{4} \end{array}\right]=\left[\begin{array}{c} h\left(y_{3}\right)-y_{1}\\ h\left(y_{4}\right)-y_{2}\\ -y_{3}-h\left(y_{4}\right)\alpha -y_{1}\beta +1\\ -y_{4}-h\left(y_{3}\right)\alpha -y_{2}\beta +1+c\left(t\right)\lambda \end{array}\right], \end{array}$$

(2) $$\begin{array}{l} h\left(z\right)=\max\left(z,0\right), \end{array}$$

where the parameters α = 2.5, β = 2.5, τ = 0.5, and γ = 0.25 define the limit-cycle **Y** ⊂ ℝ^4^ to which **y** converges; and the parameter λ = 0.5 scales the input signal *c*(*t*). The input signal of the sensory CPG is the sensory signal *c*(*t*) = *x*(*t*); thus, the limit-cycle **Y** represents the sensory phase.

### 4.2. Sensory Event Mistiming Detection

**Figure 4:**
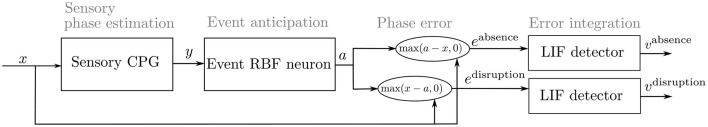
The architecture of the proposed mistiming detector with the sensory phase estimator. The sensory CPG synchronizes the sensory signal *x* and thus estimates the sensory phase ϕ^*x*^. The RBF neuron learns the phase during which the event occurs; the RBF neuron is active, *a* ≈ 1, during the anticipated event. A difference between the RBF neuron activation and sensory signal gives two types of mistiming error: *e*^absence^ and *e*^disruption^. Each error excites its respective LIF neuron, where each LIF neuron learns the activation threshold during the regular motion. If the sensory signal contains disturbances, the LIF activation *v* exceeds the threshold and fires. The LIF firing detects the mistiming.

The mistiming detection module, depicted in Figure 4, is composed of the CPG estimating the sensory phase, Radial Basis Function (RBF) neuron estimating the sensory event, and Leaky-Integrate-and-Fire (LIF) neuron, which fires on the integrated mistiming error. For each sensory input, the detector is trained to recognize two types of mistiming error: the sensory event absence and disruption.

Event mistiming occurs when a sensory event unexpectedly transpires, or no event happens when the sensory phase estimator expects it. The phase estimation is provided by the sensory CPG entrained by its respective sensory signal ${ẏ}_{i}^{\text{sense}}=f\left({\text{y}}_{i}^{\text{sense}},x_{i}\left(t\right)\right)$. Assuming the natural CPG frequency and gait frequency are similar ω^cpg^ ≈ ω^gait^, the CPG synchronizes to the sensory signal and thus estimates the phase of the sensory signal continuously.

The sensory event phase estimation is utilized by the RBF neuron, which learns to anticipate the sensory event, when *x*(*t*) ≈ 1. The RBF neuron activity coupled to the CPG represents a particular phase interval, be it motor phase (Pitchai et al., 2019) or sensory phase. The RBF neuron uses the activity function

(3) $$\begin{array}{l} \varphi \left(\text{y};\text{m}\right)=\exp\left(-\varepsilon \|\text{y}-\text{m}{\|}^{2}\right), \end{array}$$

where **y** is the CPG state and **m** is the center parameter. Hence, the RBF neuron is excited if the CPG state is near the RBF center. The excitation timing is learned to be the same as the timing of the regular sensory event using the periodic Grossberg learning rule ṁ_*i*_ = ν(*t*)*x*_*i*_(*t*)(**y**_*i*_ − **m**_*i*_). The periodic Grosberg rule pushes the RBF center near the point on the CPG limit cycle ${\text{Y}}_{i}^{\text{sense}}$ that represents the phase during the signal event *x*_*i*_(*t*) ≈ 1. Therefore, the RBF activation $\varphi \left({\text{y}}_{i}^{\text{sense}}\left(t\right);{\text{m}}_{i}^{\text{sensor}}\right)=a_{i}\left(t\right)$ anticipates the binary sensory event *x*_*i*_(*t*) ≈ 1.

Motion disturbances can perturb the timing of the sensory event. Then, the perturbed sensory event does not overlap the imitated event |*a*_*i*_(*t*) − *x*_*i*_(*t*)| > 0 and thus generates the phase error. Two types of mistiming errors are used to measure the lack of overlap: the *disruption error* (4) and *absence error* (5):

(4) $$\begin{array}{l} e_{i}^{\text{disruption}}\left(t\right)=h\left(x_{i}\left(t\right)-a_{i}\left(t\right)\right), \end{array}$$

(5) $$\begin{array}{l} e_{i}^{\text{absence}}\left(t\right)=h\left(a_{i}\left(t\right)-x_{i}\left(t\right)\right). \end{array}$$

The disruption error is non-zero $e_{i}^{\text{disruption}}\left(t\right)>0$ when the RBF neuron does not anticipate the event occurrence, while the absence error is non-zero $e_{i}^{\text{absence}}\left(t\right)>0$ when the event is anticipated but does not occur.

The mistiming errors indicate the phase perturbation; however, they can also be non-zero during the regular motion in practice. In particular, since the waveforms of the signals *a*_*i*_(*t*) and *x*_*i*_(*t*) are generally different; thus, there is always some mistiming error even during the regular motion. Moreover, false sensory events may occur due to sensory processing or measurement imperfections. Hence, in practice, the integral of the mistiming error (i.e., the absence or disruption) over one gait period $E\left(\tau \right)=\int_{\tau -T^{\text{gait}}}^{\tau }\;e\left(t\right)dt$ might be non-zero even during the regular gait, *E*(τ^regular^) > 0. We assume that if the motion is disturbed during the gait, the integrated mistiming error is greater than the regular error *E*(τ^disturbed^) > *E*(τ^regular^). Therefore it is possible to set the threshold θ = *E*(τ^regular^) which delimits the regular sensory input error from irregular.

We propose approximating the integration with the LIF neuron and adapting the firing threshold θ using a learning rule. The LIF neuron with activation dynamics ${\dot{v}}_{i}=-v_{i}\gamma +e_{i}$ fires when the neuron activation *v*_*i*_ reaches the threshold θ_*i*_. Since the threshold depends on many factors, such as the sensory variance and the shape of the CPG limit-cycle, the threshold must be parameterized for each sensory input *x*_*i*_. A similar LIF threshold parametrization problem is described in Diehl and Cook (2015), where authors introduce a learning rule for threshold adaptation. The adaptation mechanism increases the threshold during LIF firing and then slowly decays when LIF is at a non-firing activity. The LIF fire rate is then lower, and it is more likely that LIF fires at an irregular input. We employ the idea of the threshold adaptation in the following dynamics:

(6) $$\begin{array}{l} {\dot{\theta }}_{i}=\nu \left(t\right)\left(h\left(v_{i}+\gamma -\theta_{i}\right)-\left(\theta_{\text{min}}-\theta_{i}\right)\right), \end{array}$$

where γ adds margin to the threshold and θ_min_ sets the default threshold value. The threshold is adapted only during learning ν(*t*) > 0, when LIF is fed by a regular input; therefore, the LIF threshold is adapted to regular integrated phase error. For each *i*-th signal input, there are two LIF neurons. The first is for the disruption error $v_{i}^{\text{disruption}},\theta_{i}^{\text{disruption}}$ and the second is for the absence error $v_{i}^{\text{absence}},\theta_{i}^{\text{absence}}$. If a motion irregularity occurs, the integrated mistiming error (the absence or disruption) in the LIF neuron exceeds the respective threshold θ_*i*_, and the neuron fires. Thus, the firing activity of the LIF neuron *v*_*i*_ indicates the mistiming detection, which can trigger a reflex reaction modifying the regular motor control.

### 4.3. Amplitude Motor Control

The amplitude controller generates a control signal combining the regular gait motion, which produces the tripod gait, and the reflexive motion triggered by sensory event mistiming. The regular motion of an actuator is divided into four phases: first, the (i) early and (ii) late swing phases, and then the (iii) early and (iv) late stance phases, illustrated in Figure 2. Each phase defines the joint angle and torque limit set into the actuator during the motion. If a disturbance is detected, the respective reflex reaction modifies the joint angle and torque limit for a short period. Hence, the modification of the regular control causes a reflex behavior.

#### 4.3.1. Control of Regular Motion

The regular motor phase of the *j*-th actuator is generated by the motor CPG

(7) $$\begin{array}{l} {ẏ}_{j}^{\text{motor}}\left(t\right)=f\left({\text{y}}_{j}^{\text{motor}},c_{j}^{\text{motor}}\left(t\right)\right). \end{array}$$

**Figure 5:**
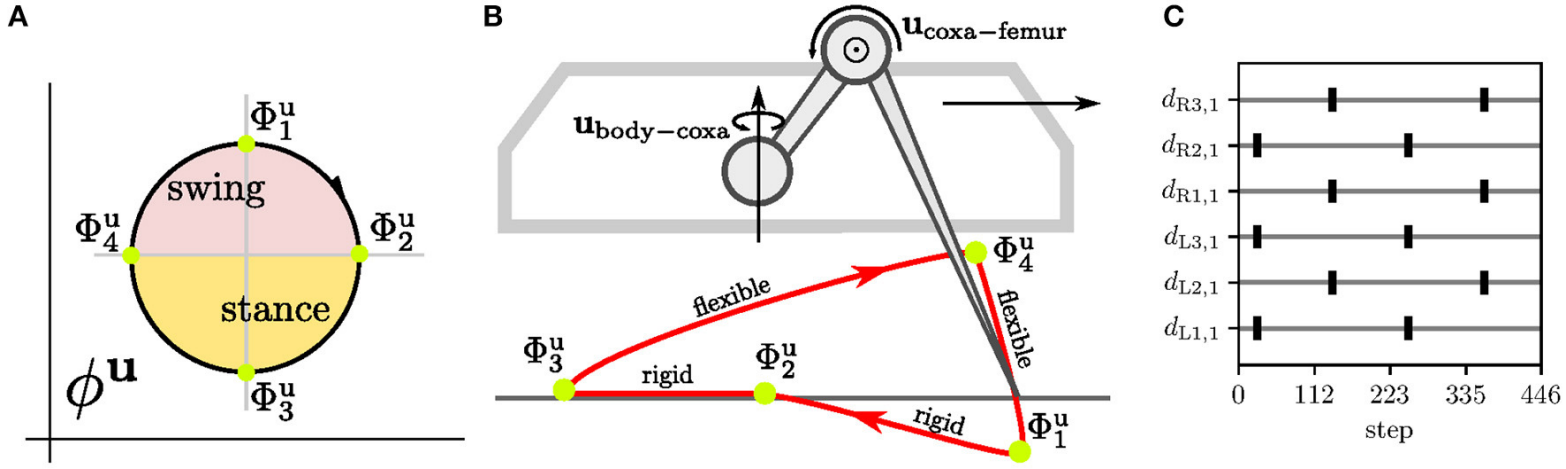
The leg motion control and the inter-limb synchronization for the tripod gait. **(A)** For each *j*-th joint, the motion is divided into four phases $\Phi_{j,1}^{u},\Phi_{j,2}^{u},\Phi_{j,3}^{u},\Phi_{j,4}^{u}$. **(B)** At the *k*-th phase, the *j*-th joint is controlled by the set control command *u*_*j,k*_ that sets the joint angle $u_{j,k}^{\text{angle}}$ and torque $u_{j,k}^{\text{torque}}$. In effect, the leg performs the motion with the foot-tip trajectory. The leg is rigid (high maximum torque set on joints) during stance so it can propel the body forward, while during the swing, the leg is flexible (low maximum torque) and stops on the obstacle contact. The contact is detected as the difference between the expected and measured positions. The ground contact is measured by poking the end of the swing at $\Phi_{j,1}^{u}$ when the flexible leg tries to lower the foot tip below the expected ground. **(C)** The relation between motion phases of each leg depends on the gait. During the tripod gait, two groups of legs move together, where the first group is composed of the left front/hind (L1, L3) and right middle leg (R2), and similarly the second with legs R1, R3, and L2. The phase relations for the tripod gait is trained by the target signal *d*. Targets for the *l*-th leg's coxas *d*_*l*,1_ representing motor phase $\Phi_{l,1}^{u}$ are shown in the plot. A single gait cycle is 223 steps long.

Four motor RBF neurons are trained with periodic Grossberg rule to be excited at the corresponding *k*-th motor phase $\Phi_{j,k}^{\text{u}}$, see Figure 5A. For the training, we generate *target binary signals* *d*_*j, k*_(*t*) ∈ [0, 1] for six-legged robot walking a tripod gait, where two tripplets of legs alternate in stance. Thus, four motor phases *k* ∈ {1, 2, 3, 4} and legs of the first group *j* ∈ {actuators of the left front/hind and right middle legs}, the signals are defined as

$$d_{j,k}(t)=\{\begin{array}{l} 1\text{ if for any }n\in \mathbb{N}:t\in [(n+(k-1)/4)T^{gait},\\ (n+(k-1)/4+0.05)T^{gait}],\\ 0\text{ else}. \end{array}$$

The target signals for actuators of the second group *j*′ are shifted $d_{j^{\prime },k}\left(t\right)=d_{j,k}\left(t+T^{\text{gait}}/2\right)$. The four motor phases on the limit-cycle ${\text{Y}}_{j}^{\text{motor}}$ are approximated by four RBF centers learned with the periodic Grossberg rule ${ṁ}_{j,k}^{\text{motor}}=\nu \left(t\right)d_{j,k}\left(t\right)\left({\text{y}}_{j}^{\text{motor}}-{\text{m}}_{j,k}^{\text{motor}}\right)$. During the learning, the motor CPG is entrained by the first target signal $c_{j}^{\text{motor}}\left(t\right)=d_{j,1}\left(t\right)$ to keep the limit-cycle consistent through multiple learning episodes; see Figure 5C. After the learning, the RBF activities $a_{j,k}^{\text{motor}}=\varphi \left({\text{y}}_{j}^{\text{motor}};{\text{m}}_{j,k}^{\text{motor}}\right)$, see (3), generate peaks, where each peak indicates the particular motor phase $\Phi_{j,k}^{\text{u}}$.

The regular motor control transforms the motor phase into regular actuator commands, see Figure 3. Commands of each *j*-th actuator are $u_{j}^{\text{angle}}=\overset{K=4}{\underset{k=1}{\sum }}a_{j,k}^{\text{motor}}u_{j,k}^{\text{angle}}$ and $u_{j}^{\text{torque}}=\overset{K=4}{\underset{k=1}{\sum }}a_{j,k}^{\text{motor}}u_{j,k}^{\text{torque}}$ for joint angle and maximum torque, respectively; where $u_{j,k}^{\text{angle/torque}}$ are the set parameters. The motion command parameters are set up so that the leg performs stance and swing, depicted in Figure 5B. The swing is designed to be flexible and protracts the leg over the ground. If the leg hits an obstacle, the leg stops due to its flexibility caused by a low torque limit. On the other hand, during the stance, the leg becomes rigid and pushes the body forward by retracting the leg. Three legs move together during the stance, the ipsilateral front, hind legs, and the contralateral middle, creating the tripod gait.

#### 4.3.2. Control During Irregular Motion

The controller provides two mechanisms reacting to the phase error: sensory-motor phase difference stabilization and reflexes. The phase difference stabilization (introduced in the base work Szadkowski and Faigl, 2020) couples the sensory and motor CPGs using a layer of sensory RBFs. Each motor CPG is connected to all sensory CPGs through RBF neurons, each trained by the target signal *d*_*j*, 1_(*t*) to find the corresponding phase on the sensory CPG. Effectively, each sensory RBF center encodes the phase difference between the particular sensory CPG and motor CPG. The averaged sensory RBF activity entrains the motor CPG, and thus the sensory-motor phase difference is stabilized.

The sensory-motor phase difference stabilization is used to handle the long term phase errors. However, reflexes represent a more suitable tool for critical errors since they affect the amplitude control by modifying the regular commands; thus, creating the reflexive behaviors. Two reflexes are implemented in this work: the search reflex and the elevator reflex. The search reflex is triggered by the absence of the ground contact event, and its reaction is the leg's rapid elevation and protraction.^1^ The elevator reflex is triggered by a disruption of the protraction stop event, where the leg rapidly retracts and elevates, and then continues the protraction. Both reflexes utilize the presented sensory event mistiming detection and demonstrate the proposed approach in a practical deployment from which results are reported in the next section.

## 5. Deployment and Empirical Validation

**Figure 6:**
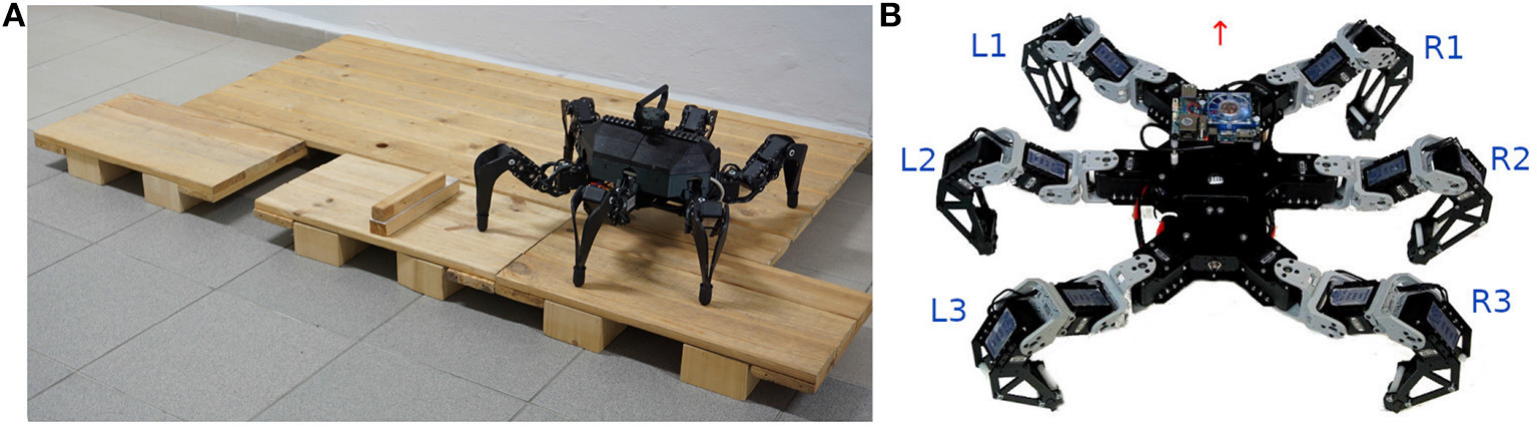
**(A)** Photo of the hexapod walking robot in the laboratory test track. The robot has six legs, each comprising three Dynamixel AX-12 servomotors; however, only the body-coxa and coxa-femur servomotors are controlled in experiments presented in this work. The servomotors also provide the joint angle measurement, which is further processed into swing stop and ground contact events for each leg. **(B)** Leg schema.

The proposed CPG-based controller has been deployed on the real hexapod walking robot depicted in Figure 6A. The setup of the deployment is detailed in section 5.1. The robot controller learns the motor control for the tripod gait and the mistiming detector; see the description provided in section 5.2. The trained controller has been examined in two scenarios. Section 5.3 reports on the first scenario, where the robot encounters two obstacles, detects mistiming events, and performs the elevator and search reflexes. The robustness of the proposed controller has been examined in the second scenario, described in section 5.4, in which the robot traverses highly unstructured terrain in the Bull Rock cave system. Further, the found insights are discussed in section 6.

### 5.1. Setup and Deployment

The proposed mistiming detector is deployed on the hexapod walking robot shown in Figure 6, a six-legged robot where each leg is formed from three Dynamixel AX-12 servomotors (Faigl and Čížek, 2019). In this work, we control two servo motors per leg: the body-coxa and coxa-femur joint servomotors; the third servomotor, femur-tibia joint, is set to a static angle. The servomotors provide the joint angle measurements processed into sensory signals for leg protraction stops and ground contact events. Both events occur during the swing when the leg is flexible. The stop of the *l*-th leg protraction $x_{l}^{\text{stop}}$ occurs at $\Phi_{4}^{\text{u}}$ (see Figure 5B), where the body-coxa servomotor position change is near zero. If the leg encounters an obstacle, the body-coxa stops sooner due to low torque. The ground contact of the *l*-th leg $x_{l}^{\text{contact}}$ occurs at the end of $\Phi_{1}^{\text{u}}$, where the coxa-femur servomotor cannot lower the leg anymore because of the ground, and the position error therefore grows. On the other hand, if there is a depression in the ground, the coxa-femur servomotor continues to lower the leg, and the contact event occurs later than usual, or not at all if the leg does not reach a foothold. Each leg generates a pair of sensory signals, $x_{l}^{\text{stop}}$ and $x_{l}^{\text{contact}}$, fed into the controller during both phases: the learning and deployment.

The dynamics of the proposed controller described by the differential equations are numerically solved by the Euler method with the step size of 0.01. The execution of 100 steps was measured to be 5.15s long (*T*^g*ait*^ = 223 steps ≈ 11.5s).

### 5.2. Tripod Gait Training and Mistiming Detection Learning

The controller has been learned in two parts with the hexapod walking robot on flat ground. First, the robot is trained to generate the motor phase. In the second part, the robot learns to detect sensory mistiming. The reflexive behavior is turned off during the learning. The individual training parts are detailed as follows.

#### 5.2.1. Tripod Gait Training

**Figure 7:**
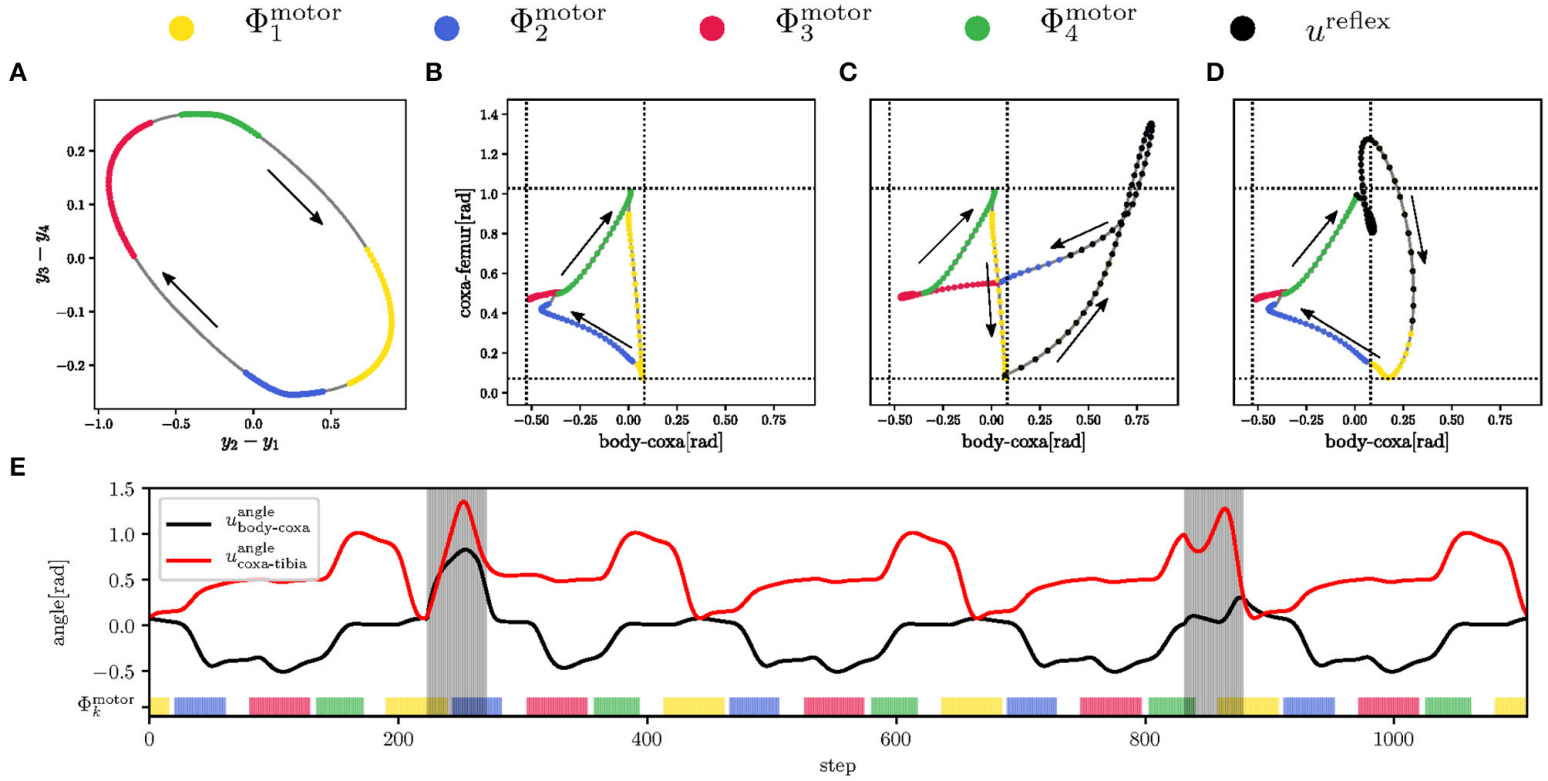
The regular and reflex motions of the left front leg during late swing $\Phi_{1}^{\text{motor}}$ (in the yellow), early stance $\Phi_{2}^{\text{motor}}$ (in the blue), late stance $\Phi_{3}^{\text{motor}}$ (in the red), and early swing $\Phi_{4}^{\text{motor}}$ (in the green). **(A)** The limit cycle *Y*^motor^ generated by the motor CPG of the front left body-coxa joint. The duration of each motor phase $\Phi_{i}^{\text{motor}}$ is projected on the limit cycle, which trajectory direction is indicated by black arrows. The motion phases determine the joint angle control. **(B)** The regular triangular leg trajectory. At the end of the late swing $\Phi_{1}^{\text{motor}}$, the leg pokes the ground. **(C)** The search reflex triggered at the end of the late swing. The leg tries to grasp for support in the protraction direction. **(D)** The elevator reflex triggered shortly after early swing $\Phi_{4}^{\text{motor}}$. The leg avoids the obstacle from above. **(E)** Five gait-cycles of body-coxa (black curve) and coxa-tibia (red curve) joint angles during regular motion and the search and elevator reflexes. Both reflexes are highlighted by the gray area, where the search reflex starts at 222 step, and the elevator reflex starts at step 832.

The motor phase generation has been trained for 30,000 steps on a flat terrain by the given target signal **d** for each joint, as shown in Figure 5C. Four motor RBFs are trained to be active during their respective motion phases, which determine the hand-tuned configuration of the control commands, see Figure 7A. The regular control signal *u*^regular^ for body-coxa and coxa-femur joint angles, shown in Figure 7B, follows the general foot-tip trajectory depicted in Figure 5B. The maximum torque *u*^torque^ is set to 1.25 N m (rigid) during stance and 0.5 N m (flexible) during swing. The reflex control signal *u*^reflex^ is hand-tuned to perform the elevator and search reflexes, plotted in Figures 7C,D, respectively. The example of joint angle evolution is shown in Figure 7E, where both reflexes occur within five gait-cycles. During any reflex, the coxa-femur servomotor, affecting the leg elevation, is rigid, while the body-coxa servomotor is flexible. The inter-leg phase relations given by the target **d**(*t*) are learned by the motor phase generator, and the hexapod robot walked the tripod at the end of the gait training. The walking hexapod robot interacts with the environment that generates the regular sensory signal, which trains the mistiming detector.

#### 5.2.2. Mistiming Detection Self-Learning

The mistiming detection is learned during 13000 steps of walking tripod gait in the regular environment, as shown in Supplementary Video 1.

**Figure 8:**
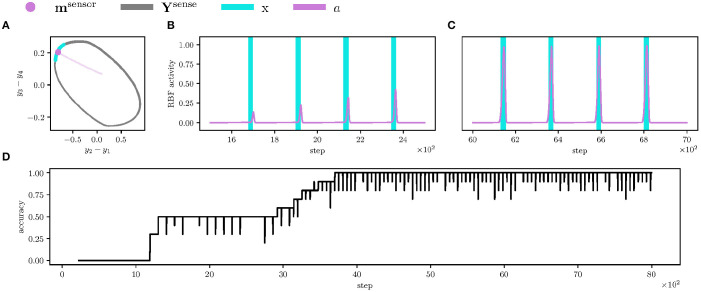
Detail of learning the left leg's contact event anticipation and the overall anticipation accuracy. **(A)** Projected CPG limit-cycle *Y*^sense^ (in the gray) and the event RBF weight *m*^sensor^ trajectory (in the magenta) of the front left leg's contact event. During the learning, the RBF weight approaches the limit-cycle segment, during which the left leg senses contact *x* > 0 (in the blue). At the end of the learning, the RBF weight (the magenta dot) is close to the limit-cycle segment; therefore, the RBF activity *a* spikes during the phase segment can be seen in the following plots. **(B)** At the start of the learning, the RBF activity *a* (in the magenta) is low and peaks outside of the left leg contact event *x* > 0 (in the blue). **(C)** However, at the end of the learning, the RBF activity peaks are close to the maximum possible activity (one), and the peaks overlap with the events. Ideally, the total number of such overlaps during one gait-cycle is twelve, one per each sensory input. **(D)** The plotted sum of the anticipation-event overlaps over a sliding window of the size *T*^gait^ = 223 divided by the number of sensory inputs (12). At step 4, 000, all RBF neuron anticipations overlap with the measured sensory events.

We first let the robot learn to anticipate the sensory events for 8,000 steps with the learning rate ν(*t*) linearly decreasing from one to zero. As can be seen in Figure 8, the event RBF neurons find their respective phase represented by a limit-cycle **Y**^sense^. At the end of the anticipation learning, the event RBF neurons anticipate the sensory events with high accuracy, as shown in Figure 8D.

**Figure 9:**
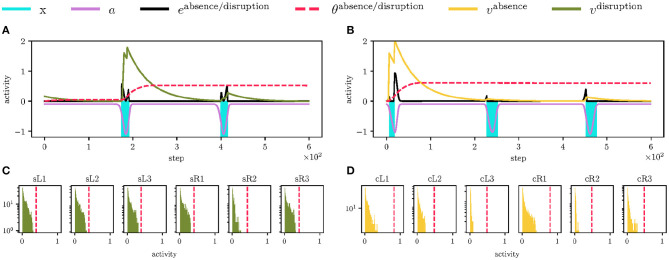
Adaptation of the firing threshold θ. **(A)** Detail of the LIF threshold θ^disruption^ (visualized as the red dashed line) adaptation for the left leg's early swing stop. Initially, the threshold is set to zero, thus LIF fires (in the green) at the first non-zero error *e*^disruption^ (in the black), where the error is rectified difference between the early stop event *x* (in the blue) and RBF anticipation *a* (in the magenta), *h*(*x* − *a*). During the LIF firing, the threshold rapidly grows; therefore, the next LIF non-zero activity at step 400 is below the threshold, and LIF does not fire. The threshold slowly decays (not observable in plots). **(B)** The LIF detector (in the yellow) for the left leg's contact absence behaves similarly. The last thousand steps of the LIF neuron activations are aggregated in histograms, where it is shown that the respective thresholds are upper-bound of the regular activations. **(C)** The swing stop perception is precise during the regular motion; thus, the LIF activity (in the green) is similar for all legs, and so are the thresholds (showed as the red dashed line). **(D)** However, the ground contact perception differs for each leg (probably due to different loads on the legs during the stance) and is less precise (the leg sometimes did not detect the ground contact). It resulted in the increased variance of the ground contact absence thresholds across the legs. Note that the contra-lateral legs (e.g., cL1 and cR1) have similar thresholds.

After the event anticipation learning, the robot adapts the LIF thresholds during 5,000 steps, where the learning rate ν(*t*) linearly decreases from one to zero. At the start, mistiming error causes LIF to fire, as it is shown in Figures 9A,B, which increases the threshold with dynamics (6). Then, the threshold slowly decays. On some occasions, the threshold descends too close to the regular LIF activity and fires again, increasing the threshold. However, since the learning rate ν(*t*) converges to zero, the threshold increments are smaller as the learning progresses.

At the end of the learning, the thresholds are adapted so LIFs do not fire in the regular environment, see Figures 9C,D. The thresholds are also close to the LIF activity maxima; therefore, LIF fires and detects the phase mistiming if there is more error accumulated due to the motion disturbances.

### 5.3. Walking Over Obstacles

**Figure 10:**
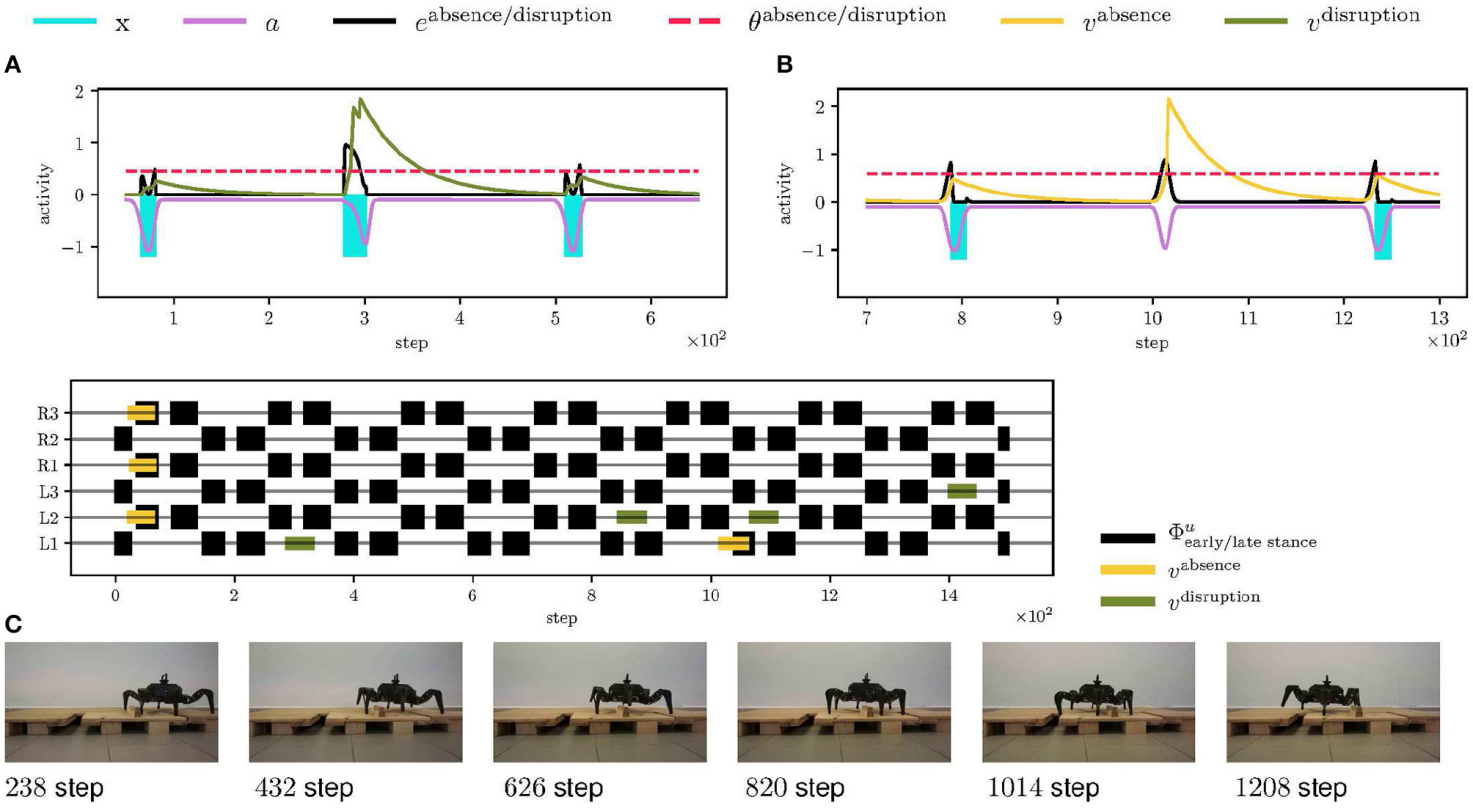
Walking over obstacles deployment scenario. **(A)** At step 300, the front left leg (L1) encounters an obstacle, which stops the swing sooner, and thus the *x*^stop^ event starts sooner, creating a high error *e*^disruption^ (in the black). The error excites the LIF neuron activity (in the green) over the threshold (visualized as the dashed red line), thus the LIF fires triggering the elevator reflex on the L1 leg. **(B)** At step 1,000, the RBF neuron anticipates ground contact, which does not happen. The absence of the error excites the LIF neuron (in the yellow) and triggers the search reflex. **(C)** An overview of the triggered elevator (in the green) and search (in the yellow) reflexes for each leg. The black events show early and late stance phases. The left legs of the hexapod walking robot gradually detect and avoid the obstacle. At step 1,050, the front left leg steps into the depression, and the search reflex is triggered. Since there are no obstacles on the robot's right side, no reflexes are triggered for the right legs.

The proposed mistiming detection is demonstrated in the deployment of the robot on track depicted in Figure 6A, where the mistiming detector triggers reflexes. The robot's left legs must negotiate one obstacle and one depression to continue its gait. The obstacle is 7 cm high and 4 cm long, which is higher than the maximum elevation during the regular swing. Hence, the leg is stopped by the swing, and the event disruption is detected, which triggers the elevator reflex, see Figure 10A. After avoiding the obstacle, the leg encounters a depression 10 cm deep, and 5 cm long, which is further than the leg reaches during regular motion. Since the leg is not stopped by the ground as anticipated, an absence of the ground collision is detected, which triggers the search reflex, see Figure 10B. The searching leg grasps the far away support, and the motion continues. In Figure 10C, we can see the right legs moving regularly as no obstacle was detected. The record of the robot walking over obstacles is provided in Supplementary Video 2.

### 5.4. Irregular Locomotion in Bull Rock Cave

**Figure 11:**
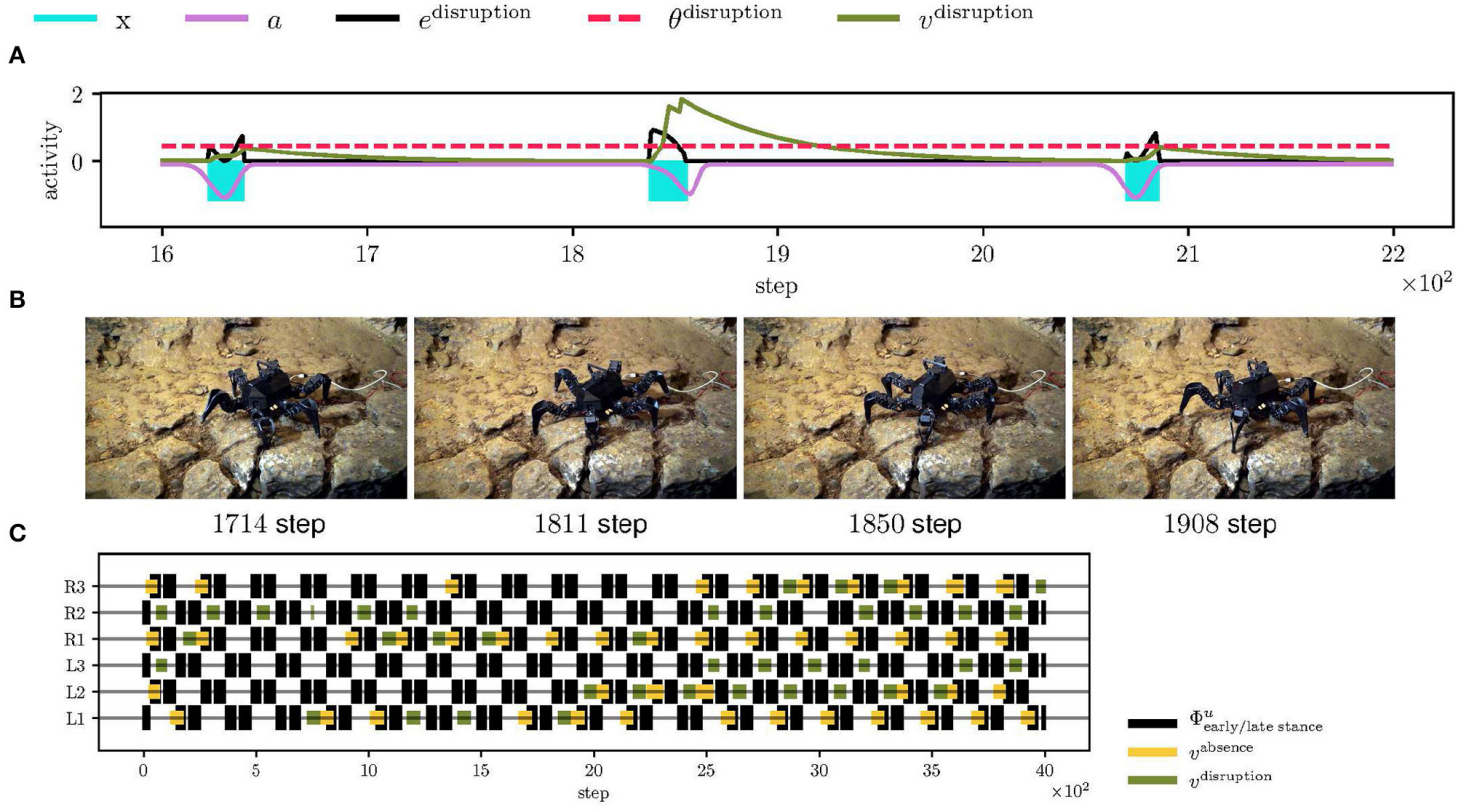
The hexapod walking robot deployed in the Bull Rock cave. **(A)** During the traversal, the front left (L1) leg got stuck in a crack for two gait-cycles. At step 1,850, the leg detects the swing stop disruption and performs the elevator reflex. **(B)** The elevator reflex worked well in this context and successfully freed L1. **(C)** An overview of the triggered reflexes. In the examined unstructured environment, the motion was highly irregular, which resulted in many triggered reflexes.

Limits of the proposed controller have been tested during the field deployment in Bull Rock cave, where the robot crawled over highly unstructured terrain with a wet slippery surface and cracks, see Figure 1A. In such an environment, multiple reflexes are triggered at once; see Figure 11C and Supplementary Video 3, which changes the locomotion of the whole body and, in some cases, detects event mistiming when there is seemingly none. For example, the combination of triggered reflexes toggles the robot on the left side, and thus when the right leg enters the stance, it touches the ground later, which triggers the search reflex. On the other hand, the elevator reflex works in unintended situations, that have been observed for a leg is stuck in a crack, which is documented in Figures 11A,B. In such a situation, the leg does not move during the swing, and thus the elevator reflex is triggered, which frees the leg. Overall, the hexapod walking robot with the proposed locomotion control traversed the highly irregular terrain multiple times and detected parallelly multiple phase mistiming, supporting the expected advantage of the mistiming detector in a real cave environment.

## 6. Discussion

The proposed controller has been trained to perform the tripod gait. During the tripod gait on flat terrain, the hexapod walking robot learned to anticipate the ground contact and swing stop with accuracy shown in Figure 8. LIFs then adapt the regular difference between sensory anticipation and measurement. The thresholds are upper-bound of the regular LIF activity, see Figure 9; therefore, LIFs are at rest during regular motion. The benefit of mistiming detection is further demonstrated in two deployment scenarios where mistiming detection triggers the designed reflex reactions. The reflexes allowed the robot to locomote through terrains that are otherwise untraversable with the regular gait. From this perspective, the expected advantage of the proposed idea has been fulfilled.

On the other hand, in some cases, the reflexes were triggered even though there was no obstacle nor depression. In the testbed scenario visualized in Figure 10C, the middle left leg performs the elevator reflex at step 1100, albeit the leg already cleared the obstacle at step 900. The elevator reflex at step 1,100 has been triggered by detected early swing stop, which has not been caused by an obstacle, but by the search reflex of the front left leg triggered at step 1,050. Such behavior can also be observed in Figure 11C, where the search reflex of the front legs causes the elevator reflex of the middle legs. The search reflex leaves the robot body slightly tilted, which causes the adjacent middle leg to stop the swing earlier. Thus, the middle left leg detects the search reflex of the adjacent leg. It is a cautionary tale that the interpretation of mistiming detection, or generally any sensory error, is dependent on the context in which the robot is. The direct interpretation of the situation in which an obstacle stops the swing is correct only if the robot's current state is close to the state of the regular motion. Sustaining the regular gait motion improves not only the locomotion but also the interpretability of the sensory input. Therefore, improving the gait control, e.g., adding balancing reflex, is one strategy preventing incorrect interpretation of the sensory input. Another strategy can be based on fusing multiple sensory inputs as it is less likely that each of the sensory input provides incorrect interpretation at the same time.

The proposed mistiming detector relies on the CPG providing the sensory phase estimation; thus, the mistiming detector inherits the robustness of the CPG dynamics but also its drawbacks. While short-term changes of sensory signal properties have little effect on the CPG, if the change is lasting, then the CPG behavior changes as well. Consider that the sensory signal changes in phase or frequency. If the sensory signal changes in phase, the sensory CPG shifts its phase and maintains the stable phase difference between the signal and the CPG. However, there are more possible outcomes if the sensory signal frequency of ω^*c*^ changes. The CPG has a range of detuning Δω = ω^*c*^ − ω^cpg^ where the CPG can synchronize with the input signal (Pikovsky et al., 2001). Outside the synchronization range, the phases of the CPG and input signal evolve with different speeds; therefore, if the detuning is too high ^2^, the sensory CPG does not estimate the sensory phase.

In the gait control context, the sensory inputs for the mistiming detector are a consequence of the interaction between the environment and periodic motor activity. A persistent change in motor activity can induce a change in the sensory signal, influencing the sensory CPGs, as described above. The terrain in Bull Rock cave is a source of such persistent change, see Figure 11, where the rough terrain caused a change in the motor activity by triggering one reflex after another. Although it was not observed during the short span of the Bull Rock cave deployment, the change of the sensory CPG properties (phase or frequency) influences the motor phase generation (see Figure 3), which may compromise the gait pattern. Therefore, the presented gait controller can generate a disturbed motion pattern if it operates in a highly unstructured environment. Such disturbances can be prevented by adding more reflexes, which would stabilize the regular motion, or the controller can react to an unstructured environment by a switch to a different gait. For both cases, the mistiming detector provides the means to recognize a highly irregular environment.

The mistiming detection adds an alternative to usual amplitude error detection, where the measured sensory value rises above some threshold. Notice, from a practical point of view, the ground contact absence and the swing stop detections are implemented simply from reading the position from the Dynamixel AX-12 servomotors, without the need for any additional sensory equipment. Generally, the proposed mistiming error augments the information gained from the measured sensory input, and further utilization of the augmentation is a subject of our future work.

## 7. Conclusion

In this paper, we present a novel learnable CPG-based event mistiming detection. We propose to combine CPG with the RBF neuron into a sensory event estimator and compare the estimation with measurement to assess the phase error. The phase error is integrated by the LIF neuron, which detects the irregularity in the timing of event occurrence. The proposed mistiming detection is self-learned with dynamic Hebb-like learning rules by the robot on which the system is deployed. We integrated the mistiming detection with the CPG-based gait controller, where the detection triggers reflexive behavior. An absence of the ground contact triggers the search reflex, while the elevator reflex is triggered by detecting an obstacle during the swing. The CPG-based controller is deployed on a real hexapod walking robot, which is trained to walk using a tripod gait and learns the properties of twelve sensory signals. The learned controller has been examined in two deployment scenarios. In the laboratory testbed, the robot encounters a depression and an obstacle on flat terrain, where each leg reacts independently with corresponding reflexes. In the second scenario, we demonstrate the robustness of the proposed controller in Bull Rock cave, where the robot traverses slippery and highly unstructured terrain. The proposed plastic CPG-based mistiming detection enhances the information gained from the periodic sensory signal, which can be utilized not only for reflex control but also can serve as an input for other control centers.

## Data Availability Statement

The raw data supporting the conclusions of this article will be made available by the authors, without undue reservation.

## Author Contributions

RS conceived and designed the study. RS and MP performed the experiments and processed the data. With the support of MP and JF, RS wrote the manuscript. All the authors contributed to the manuscript and approved the submitted version.

## Conflict of Interest

The authors declare that the research was conducted in the absence of any commercial or financial relationships that could be construed as a potential conflict of interest.

## Footnotes

^1^It is a simplified version of the search reflex observed in a locust (Pearson and Franklin, 1984), where the insect searches for the foothold with circular motions.

^2^In particular, the synchronization range depends on the input signal strength, which is set to λ = 0.5 in this work. The range gets smaller with lesser input strength creating a structure in the λ-Δω plane called the Arnold tongue. In general, the Arnold tongue cannot be found analytically, yet there must be some small synchronization region around Δω = 0 for high enough λ.

## Supplementary Material

Supplementary Video 1 The hexapod walking robot self-learns the mistiming detector.

Supplementary Video 2 The hexapod walking robot walks over obstacles.

Supplementary Video 3 The hexapod walking robot deployed in Bull Rock cave.
